# A Normal Displacement Model and Compensation Method of Polishing Tool for Precision CNC Polishing of Aspheric Surface

**DOI:** 10.3390/mi15111300

**Published:** 2024-10-25

**Authors:** Yongjie Shi, Min Su, Qianqian Cao, Di Zheng

**Affiliations:** 1 School of Intelligent Manufacturing, Jiaxing Vocational Technical College, Jiaxing 314036, China; 2 Ministry of Basic Education, Jiaxing Vocational Technical College, Jiaxing 314036, China; sum103@126.com; 3 College of Information Science and Engineering, Jiaxing University, Jiaxing 314001, China; qqcao@zjxu.edu.cn; 4 School of Mechatronics and Energy Engineering, Ningbo Tech University, Ningbo 315100, China

**Keywords:** displacement compensation, CNC polishing, aspheric surface, deformation

## Abstract

The position accuracy of the polishing tool affects the surface quality of the polished aspheric surface. The contact deformation among the polishing tool, abrasives, and aspheric part can cause a displacement, which, in turn, will cause a position error of the polishing tool, which will lead to a significant change in the polishing force. In order to resolve this error, this paper proposed a method of normal displacement compensation for a computer numerical controlled (CNC) polishing system by controlling the polishing force. Firstly, the coupling principle between the polishing force and the position of the polishing tool is expounded, and the relationship between normal displacement and deformation is analyzed. Based on Hertz’s theory, a model of normal displacement is established. Then, on the basis of the decoupled polishing system developed, a normal displacement compensation method was proposed. Finally, a group of comparative experiments was carried out to verify the effectiveness of the proposed method. Compared with no displacement compensation, when the part was polished with the normal displacement compensation method, the value of roughness decreased from 0.4 µm to 0.21 µm, and the unevenness coefficient of surface roughness decreased from 112.5% to 19%. The experimental results show that the polishing quality is improved greatly, and the aspheric surfaces can be polished more uniformly with the method proposed in this paper.

## 1. Introduction

Aspheric parts are widely used as basic components in aerospace, electronics, defense, and other fields due to their excellent optical properties [1]. At present, the industry mainly adopts grinding and polishing as its final finishing processing. With the rapid increase in the need for high-quality aspheric parts, the development of aspheric ultra-precision machining technology and equipment is becoming much more important [2], and many automatic polishing techniques have been proposed [3].

However, in the common automatic polishing system, the polishing force is generally generated and controlled by the motion of the polishing tool and the contact deformation between the polishing tool and the aspheric surface [4]. The contact deformation will lead to a normal displacement error of the polishing tool, and then the polishing force will change greatly. This means that there is a close coupling relationship between the polishing force and the tool displacement. It is difficult to ensure the uniformity of material removal, profile accuracy, and surface quality. Thus, it is very important to control and compensate for this normal displacement change so as to ensure that the polishing force does not change abruptly.

In order to solve this problem, many researchers have put forward many useful approaches [5,6]. Some scholars have studied the key technology of deterministic polishing. Zhang et al. [7] studied the process planning of the automatic polishing of the curved surface using a five-axis machining tool. Some scholars have studied the wheel polishing technology [8]. Yao et al. [9] designed a pneumatic floating structure used to compensate for the Z motion error of the robot to realize the stable control of polishing pressure based on industrial robots. Some scholars have studied ballonet polishing [10,11]. In those studies, many polishing force control devices were proposed.

On the other hand, some scholars have studied the polishing path of the polishing tool. Zhao et al. [12] proposed a revised Archimedes spiral polishing path, which is generated based on the modified tool–workpiece contact model and the pointwise searching algorithm. Han [13] proposed an adaptive polishing path optimization method based on footprint evolution, which considers the influence of curvature on footprint evolution. Qu et al. [14] proposed an optimized Archimedes spiral path to ensure uniform material removal depth in aspheric polishing. In those studies, the effect of the surface curvature variations on material removal was eliminated.

As an overview of the above research, those approaches mainly focus on force control strategies and tool path planning, but it does not take into account the effect of the normal displacement on the position and posture of the polishing tool, and there is no study taken on the normal displacement compensation. Therefore, it is necessary to study the relationship between displacement and deformation in depth and propose a displacement compensation method.

This study focused on the model and method of the normal displacement compensation based on a computer numerical controlled (CNC) machine tool. The material removal principle was introduced, and the coupling principle of polishing force and position of the polishing tool was analyzed. Then, the models of normal displacement were established, considering the contact condition among the polishing tool, part, and abrasives. Further, based on the polishing system, the normal displacement compensation method was proposed. Subsequently, the validity of the model and method is examined experimentally.

**Figure 1:**
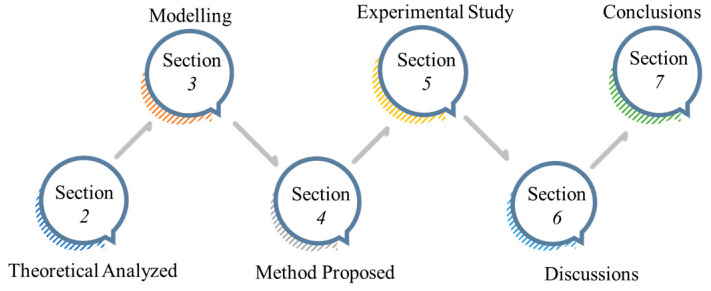
Structure of the paper.

The main structure of this paper as shown in Figure 1 is organized as follows: Section 2 derives the coupling principle of polishing force and position; Section 3 analyzes the normal displacement and establishes a normal displacement model; Section 4 proposes a polishing system and a normal displacement compensation method; Section 5 shows experiments study and gives the experimental results; Section 6 presented discussions; finally, Section 7 makes some conclusions.

## 2. Coupling Principle of Polishing Force and Position

### 2.1. Toolhead and Abrasive

The toolhead, which contacts with the aspheric surface, is a carrier for coating or embedding abrasives. Its material hardness is generally lower than that of the part, and the structure is uniform and dense. At the same time, it also has a certain abrasive embedment and immersion. The surface accuracy and retention of the toolhead affect the polishing quality. The shape of the abrasive particles is mostly irregular. In this paper, it is assumed that the particles are spherical and their shape and size are uniform.

### 2.2. Material Removal Principle

It is the result of the interaction among the toolhead, the abrasive particles, and the part that leads to tiny material removal. In this paper, soft toolhead and free abrasive polishing methods are mainly used. During the process of polishing, the abrasive is coated on the surface of the soft toolhead. The particles are free between the toolhead and the part, which means the particles have greater freedom of movement. They can be fixed and semi-fixed on the toolhead and can also slide and roll between the toolhead and the part surface.

**Figure 2:**
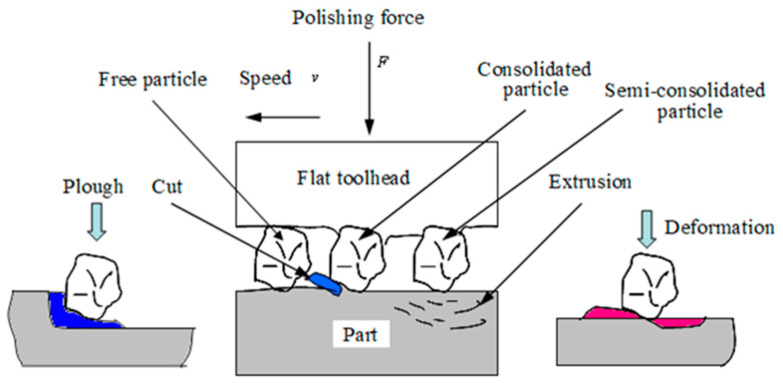
Material removal principle.

The material removal mechanism is shown in Figure 2. The whole mechanical action process is a process of extrusion, sliding, plowing, and cutting. The aspheric surface undergoes elastic deformation, plastic deformation, and micro-cutting. This is the main reason for the normal displacement of the toolhead. Compared with the wear and cutting caused by particles, the wear of the part caused by the toolhead can be neglected.

### 2.3. Force–Position Coupling Process

**Figure 3:**
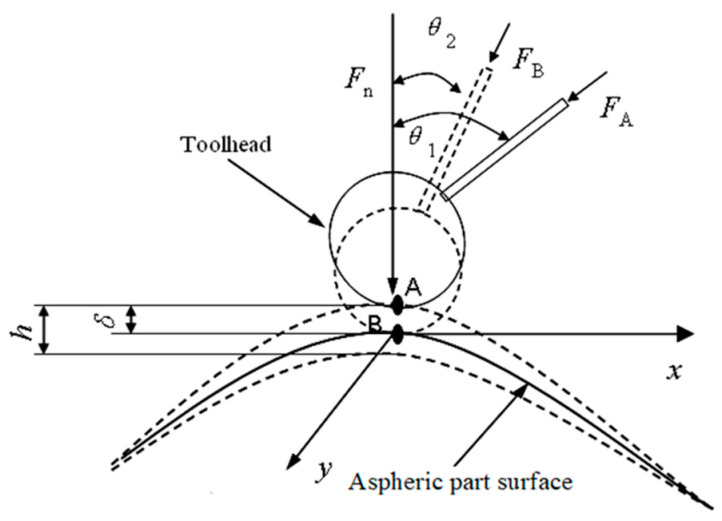
Process of force–position coupling.

Figure 3 shows the coupling process between the polishing force and the position of the toolhead. When the aspheric part is polished, in order to ensure machining accuracy, it is hoped that the part will be polished with the ideal polishing force *F*_A_, and the toolhead moves along the ideal polishing point *A* according to the pre-set trajectory, the ideal posture of toolhead can be express by *θ*_1_. However, due to the elastic–plastic deformation among the toolhead, the abrasive, and the part, there is a normal displacement *δ* of the toolhead, which will cause an error in the position of the toolhead.

It can be seen from Figure 3 that the actual position of the toolhead changes from the ideal position A to the actual position *B*, and the actual posture of the toolhead changes from *θ*_1_ to *θ*_2_. At this time, the ideal polishing force *F*_A_ and the normal polishing force *F*_An_ applied to the polishing point change into the actual values *F*_B_ and *F*_Bn_, respectively. It can be seen that due to the effect of normal displacement, the polishing force and the position of the toolhead all are changed, which leads to the polishing force and position coupling closely.

Therefore, in order to eliminate the error caused by normal displacement to ensure the quality and efficiency of the polishing, in the actual polishing, the normal displacement needs to be compensated to ensure that the actual polishing force does not change.

## 3. Modeling of Normal Displacement

During the polishing process, the normal displacement among the toolhead, the aspheric surface, and the abrasive mainly comes from contact deformation. When the toolhead and the aspheric surface contact each other, the elastic deformation occurs first. As the deformation increases, the deformation changes from elastic deformation to plastic deformation. As the residence time increases, under the action of the abrasive, micro-cutting occurs so that the aspheric surface material can be removed, and the toolhead produces a certain amount of wear.

### 3.1. Analysis of Normal Displacement Change

**Figure 4:**
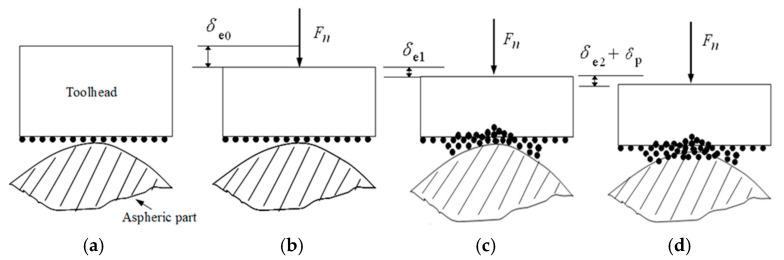
The change in normal displacement among toolhead, aspheric part, and abrasive. (**a**) Initial contact; (**b**) deformation *δ*_e0_; (**c**) deformation *δ*_e1_; (**d**) elastic–plastic deformation.

The change process of the normal displacement after the contact between the toolhead and the aspheric part is shown in Figure 4. On the contact interface composed of toolhead, abrasive, and aspheric part, the hardness of the toolhead is the lowest of the three. At the beginning of contact, the large elastic–plastic deformation and wear of the toolhead will occur. Figure 4b shows the elastic deformation *δ*_e0_ between the toolhead and the aspheric surface, and Figure 4c shows the elastic deformation *δ*_e1_ between the toolhead and the abrasive.

With the continuation of polishing, under the action of the polishing force, the depth of the abrasive cut into the aspheric surface is very small. There is only elastic deformation *δ*_e2_ occurring on the aspheric surface. Then, with the contact stress applied by the abrasive on the aspheric surface gradually increasing, elastic deformation of the aspheric surface changes into plastic deformation *δ*_p_, as shown in Figure 4d. Finally, as the depth of the abrasive cut into the surface increases, small cutting occurs, and the material is removed.

In summary, the normal displacement *δ* in the polishing process mainly includes the elastic deformation *δ*_0_ between the toolhead and the aspheric surface, the elastic deformation *δ*_e1_ between the toolhead and the abrasive, the elastic deformation *δ*_e2_ and plastic deformation *δ*_p_ between the abrasive and the aspheric surface. *δ* can be expressed as follows:

*δ* = *δ*_e0_ + *δ*_e1_ + *δ*_e2_ + *δ*_p_ (1)

### 3.2. Abrasive Distribution Model

**Figure 5:**
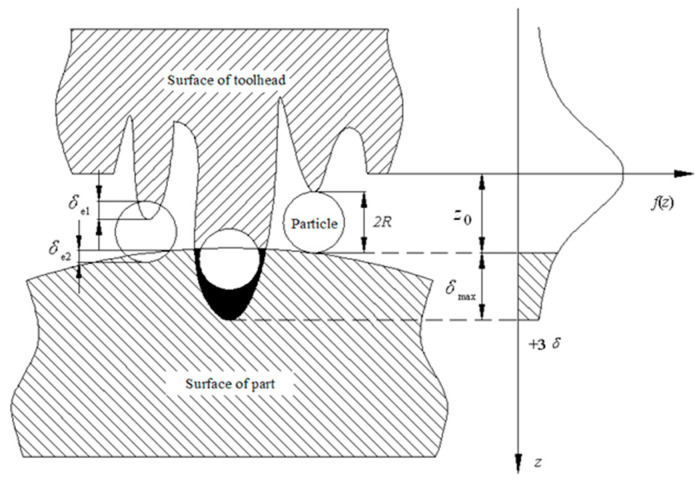
Micro-contact condition of polishing area.

Generally, it is believed that the contact between the toolhead and the part can be equivalent to the contact between a rough surface and a smooth surface. During the polishing process, the microscopic interaction among the toolhead, aspheric surface, and abrasive particle is shown in Figure 5.

It can be seen that not all particles interact with the part surface. Some particles plow or cut the part to produce the plastic deformation *δ*_p_, some particles squeeze and slip on the part surface to produce elastic deformation *δ*_e_, and some particles only roll on the part surface without any force and deformation. The number of particles per unit area *N*_0_ can be calculated as follows [15]:

(2) $$N_{0}={\left(\frac{3V_{g}}{4\pi R^{3}}\right)}^{2/3}$$

Here, *V*_g_ is the particle rate. According to the statistical model, in the small polishing contact area, the profile of the particle attached to the surface of the toolhead is approximated as follows:

(3) $$f(z)=\frac{1}{\sqrt{2\pi }\sigma }e^{-\frac{{\left(z\right)}^{2}}{2\sigma^{2}}}$$

where *z* is the protrusion height of the abrasive grain, *σ* is the standard deviation, and *f*(z) is the distribution function of particle height on the surface of the toolhead. Let z_0_ be the distance between the part surface and the reference plane. When *z* > z_0_, elastic and plastic deformation occurs among the toolhead, the abrasive particles, and the aspheric surface. Let *δ*_max_ be the maximum deformation in the interval (−3σ, 3σ). Because of $\int_{-3\sigma }^{3\sigma }f(z)dz=0.999$, the distribution function of the surface profile can be expressed as follows:

(4) $$f(z)=\left\{\begin{array}{cc} 0 & -\infty <z<-3\sigma \\ \frac{1}{\sqrt{2\pi }\sigma }e^{-\frac{z^{2}}{2\sigma^{2}}} & -3\sigma <z<3\sigma \\ 0 & 3\sigma <z<\infty \end{array}\right.$$

### 3.3. Normal Displacement Modeling

Based on the force balance among the toolhead, part, and abrasive in the polishing contact area, the deformation and the resulting displacement can be analyzed. Several assumptions are made as follows:

① The particle hardness *H*_Bm_ is higher than that of the part and toolhead, and elastic-plastic deformation between them only occurs when they are in contact with each other;
② Each particle only interacts with the part surface once at the same polishing point;
③ The contact area is very small, and all contact points within the area have the same curvature radius;
④ All particles are spherical and have the same average radius.

#### 3.3.1. Elastic Deformation

**Figure 6:**
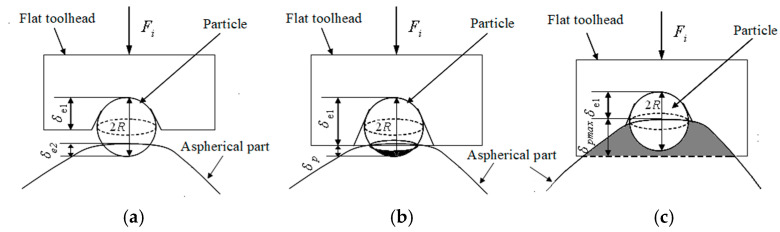
The microaction process of a single particle. (**a**) Elastic deformation; (**b**) elastic–plastic deformation; (**c**) fully plastic deformation.

The micro-contact among the toolhead, particles, and aspheric surface is shown in Figure 6.

In the initial stage of polishing, there is only elastic deformation. According to the Hertz theory [16], the elastic deformation *δ*_e0_ between the toolhead and the part can be given as follows:

(5) $$\delta_{e0}={\left(\frac{9{F_{e0}}^{2}}{16R_{\rho 0}{E_{0^{*}}}^{2}}\right)}^{1/3}$$

where *F*_e0_ is the polishing force applied perpendicularly to the part surface at the polishing point; *E*_0_* is the relative elastic modulus of toolhead and part, given by 1/*E*_0_* = (1 − *ν*_1_^2^)/*E*_1_ + (1 − *ν*_2_^2^)/*E*_2_, where *E*_1_ and *E*_2_, and *ν*_1_ and *ν*_2_ are the elastic modulus and the Poisson’s rates of the toolhead and part, respectively; *R_ρ_*_0_ is the equivalent curvature radius, because of the curvature radius of plane toolhead tends to ∞, so *R_ρ_*_0_ = *R*_e_, where *R*_e_ is the curvature radius of the part.

Figure 6a illustrates the elastic deformation among the single particle, the part, and the toolhead. The deformation *δ*_e1_ and the average pressure *P*_m1_ between the toolhead and the particle can be obtained as follows:

(6) $$\delta_{e1}={\left(\frac{9F_{i}^{2}}{16R_{\rho 1}E_{1}{*}^{2}}\right)}^{1/3}$$

(7) $$P_{m1}=\frac{1}{\pi }{\left(\frac{16E_{1}{*}^{2}F_{i}}{9{R_{\rho 1}}^{2}}\right)}^{1/3}$$

Here, *F*_i_ is the force applied to a single abrasive particle, *E*_1_* and *R_ρ_*_1_ are the relative elastic modulus and equivalent curvature radius of toolhead and particle, respectively, given by 1/*E*_1_* = (1 − *ν*_1_^2^)/*E*_1_ + (1 − *ν*_3_^2^)/*E*_3_, *R_ρ_*_1_ = *R*, where *E*_3_ and *ν*_3_ are the elastic modulus and Poisson’s rates of toolhead and particle, respectively; *R* is the radius of the particle.

Similarly, the deformation *δ*_e2_ and the average pressure *P*_m2_ between the particle and the part can be obtained as follows:

(8) $$\delta_{e2}={\left(\frac{9F_{i}^{2}}{16R_{\rho 2}E_{2}{*}^{2}}\right)}^{1/3}$$

(9) $$P_{m2}=\frac{1}{\pi }{\left(\frac{16E_{2}{*}^{2}F_{i}}{9{R_{\rho 2}}^{2}}\right)}^{1/3}$$

where *E*_2_* and *R_ρ_*_2_ are the relative elastic modulus and equivalent curvature radius of the particle and part, respectively, given by 1/*E*_2_* = (1 − *ν*_2_^2^)/*E*_2_ + (1 − *ν*_3_^2^)/*E*_3_, 1/*R_ρ_*_2_ = 1/*R* + 1/*R*_e_.

Existing studies show that when the average contact pressure *P*_m2_ ≤ *H*_Bf_/3 (*H*_Bf_ is the Brinell hardness of the part) [17], only elastic deformation occurs between the part and the particle. When *P*_m2_ = *H*_Bf_/3, the plastic flow begins in the surface layer of the part, and plastic deformation occurs. Therefore, the maximum elastic deformation *δ*_e2max_ between the part and the particle can be obtained from Equations (8) and (9):

(10) $$\delta_{e2\max}=\frac{\pi^{2}R_{\rho 2}{H_{Bf}}^{2}}{16E_{2}{*}^{2}}$$

From Equation (9), *F_i_* can be written as follows:

(11) $$F_{i}=\frac{\pi^{3}{R_{\rho 2}}^{2}{H_{Bf}}^{3}}{48E_{2}{*}^{2}}$$

Because the elastic contact force between the particle and the toolhead is equal to the plastic contact force between the particle and the part. Substituting Equation (11) into Equation (6), the maximum elastic deformation *δ_e_*_1max_ can be obtained as follows:

(12) $$\delta_{e1\max}={\left(\frac{{R_{\rho 2}}^{4}{H_{Bf}}^{4}}{16R_{\rho 1}E_{1}{*}^{2}E_{2}{*}^{4}}\right)}^{1/3}$$

Particularly, when the number of particles is large enough, there is no contact between the toolhead and the part, and the maximum normal displacement *δ*_max_ can be obtained as follows:

*δ*_max_ = *δ_e_*_0_ + *δ_e_*_1max_ + *δ_e_*_2max_ (13)

#### 3.3.2. Plastic Deformation

When *P_m_*_2_ ≥ *H_Bf_*/3, *δ_e_*_2_ exceeds the *δ*_e2max_, the plastic deformation occurs, and the particle will plow or cut the part surface. The micro-contact is shown in Figure 6b,c.

(1) *δ*_e1_ + *δ*_p_ ≤ 2*R*

Assuming that *a*_p_ is the radius of the contact zone between the particle and the part, we can obtain

(14) $$a_{p}=\sqrt{\delta_{p}(2R-\delta_{p})}=\sqrt{\left(z-z_{0})(2R+z_{0}-z\right)}$$

When *δ_e_*_1_ + *δ_p_* ≤ 2*R*, in the range of [*z*_0_ + *δ*_e2max_, *z*_0_ + 2*R*], the force *F_p_*_1_ that causes plastic deformation of part can be given as follows:

(15) $$F_{p1}=N_{0}F_{i}\int_{z_{0+\delta_{e2\max}}}^{z_{0}+2R}f(z)dz$$

In this case, the average pressure *P_m_*_2_ = *H_Bf_*/2, *F*_i_ can be expressed as follows:

(16) $$F_{i}=\pi {a_{p}}^{2}P_{m2}=\frac{\pi }{2}(z-z_{0})(2R+z_{0}-z)\times H_{Bf}$$

From Equations (15) and (16), *F_p_*_1_ can be obtained:

(17) $$F_{p1}=\frac{\sqrt{\pi }H_{Bf}N_{0}}{2\sqrt{2}\sigma }\int_{z_{0+\delta_{e2\max}}}^{z_{0}+2R}(z-z_{0})(2R+z_{0}-z)\times e^{-\frac{z^{2}}{2\sigma^{2}}}dz$$

Since plastic deformation *δ_p_* is very small, *F*_i_ can also be expressed as follows:

(18) $$F_{i}=H_{Bf}\times \pi \times R\times \delta_{p}$$

On the other hand, there is elastic contact between the toolhead and the particle. The contact force can be given as follows:

(19) $$F_{ie}=\frac{4}{3}E_{1}*R^{1/2}{\delta_{e1}}^{3/2}$$

*F*_ie_ = *F*_i_, based on Equations (18) and (19), we can obtain

(20) $$\delta_{p}=\frac{4E_{1}*R^{1/2}{\delta_{e1}}^{3/2}}{3\pi H_{Bf}R}$$

Especially when *δ_e_*_1_ + *δ_p_* = 2*R*, based on Equation (20), we can obtain

(21) $${\delta_{p}}^{3}+R(9\pi^{2}{H_{Bf}}^{2}/16E_{1}{*}^{2}-6){\delta_{p}}^{2}+12R^{2}\delta_{p}-8R^{3}=0$$

(2) *δ*_e1_ + *δ*_p_ ≥ 2*R*

When *δ_e_*_1_ + *δ_p_* ≥ 2*R*, it can be seen from Figure 6c, *a_p_* = *R*; thus, *F_i_* can be expressed as follows:

(22) $$F_{i}=\pi {a_{p}}^{2}P_{m2}=\frac{\pi }{2}R^{2}\times H_{Bf}$$

When *δ_e_*_1_ + *δ_p_* = 3*σ*, from Equation (20), *δ_p_* can be written as follows:

(23) $${\delta_{p}}^{3}+(9\pi^{2}{H_{Bf}}^{2}R/16E_{1}{*}^{2}-9\sigma ){\delta_{p}}^{2}+27\sigma^{2}\delta_{p}-27\sigma^{3}=0$$

#### 3.3.3. Normal Displacement Model

Based on the above analysis, it can be seen that during the polishing process, when the materials were removed by particles, complete plastic deformation occurs between the particles and the part, then *δ*_e2_ = *δ*_e2max_, *δ*_p_ = *δ*_pmax_ = 3*σ*. Furthermore, the normal displacement *δ* can be obtained as follows:

(24) $${\delta =\delta_{e0}+\delta_{e2max}+\delta_{pmax}=\left(\frac{9{F_{e0}}^{2}}{16R_{\rho 0}E_{0}{*}^{2}}\right)}^{1/3}+\frac{\pi^{2}R_{\rho 2}{H_{Bf}}^{2}}{16{E_{2*}}^{2}}+3\sigma$$

## 4. Polishing System and Displacement Compensation Method

### 4.1. Description of the Polishing System

**Figure 7:**
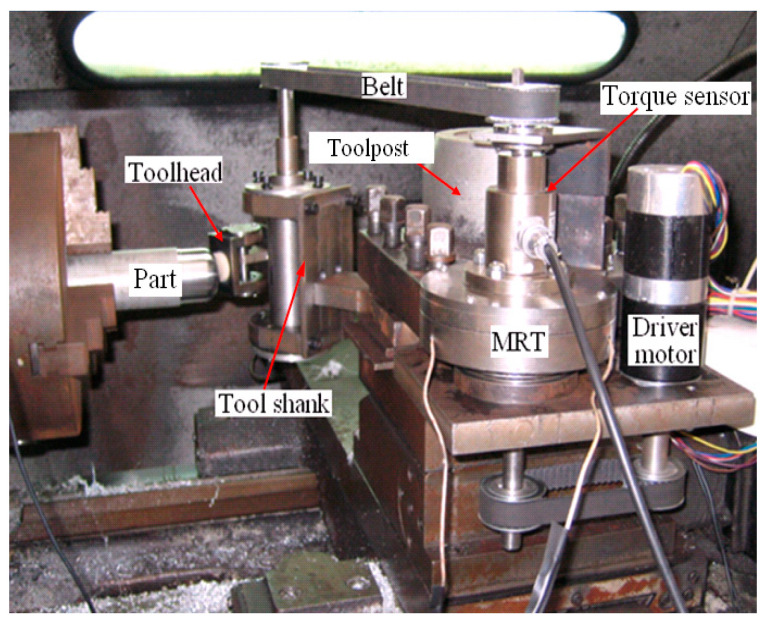
Polishing system.

A polishing system developed for NC polishing of aspheric surface is shown in Figure 7. The system mainly includes three subsystems: (1) the polishing force control subsystem based on magnetorheological torque servo device (MRT) is mainly composed of magnetorheological torque servo device, controllable current source, torque detection, and control system. During the polishing process, the polishing force is provided and controlled by the MRT, and the constant force polishing is achieved; (2) a position and posture control subsystem, mainly composed of a CNC system, polishing toolhead, transmission, tool holder, and so on. The polishing toolhead designed in this paper can adapt to the curvature variations of the part surface. During the polishing process, the CNC system controls the trajectory of the polishing tool system, and MRT and the transmission device ensure that the toolhead is always perpendicular to the aspheric surface feed; therefore, it is possible to compensate for the normal displacement of the toolhead, and the ideal polishing trajectory will not be changed; (3) the main motion subsystem, which was provided by the NC lathe to control the rotation speed of the aspheric part.

### 4.2. Magnetorheological Torque Servo Device (MRT)

**Figure 8:**
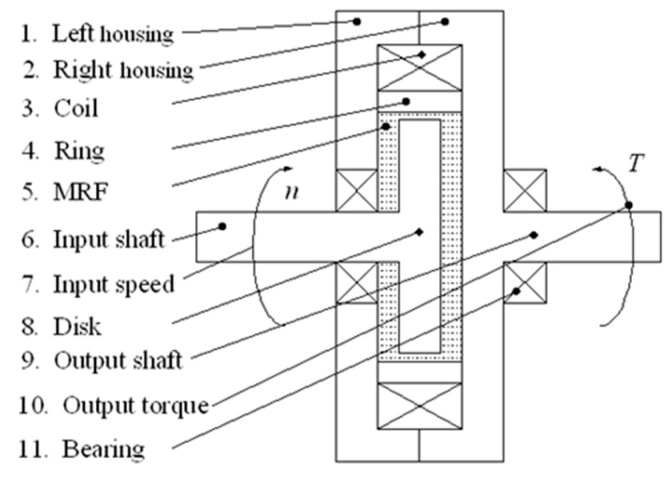
MRT structure.

The magnetorheological torque servo device (MRT) [18] developed by the authors is a force control device based on the magnetorheological (MR) effect. According to this device, the servo control of torque can be achieved. During the polishing process, the torque is converted into polishing force by means of a polishing tool system, and the servo control of polishing force can be achieved. Figure 8 shows the structure of MRT, which works in shear mode. The MR fluid becomes solidified in milliseconds when a magnetic field is applied, resulting in a shear yield stress *τ*_b_. When the working disc is driven by the input shaft, MR fluid is sheared, thereby generating an output torque. By changing the current applied to the coil of the MRT, the magnetic field strength passing through the MR fluid can be changed, and then the shear yield stress and output torque can be changed. Through experimental tests, the torque model of the MRT designed in this paper can be obtained [18]:

*T* = 1.1398*I* − 0.3812 (25)

### 4.3. Displacement Compensation Method

**Figure 9:**
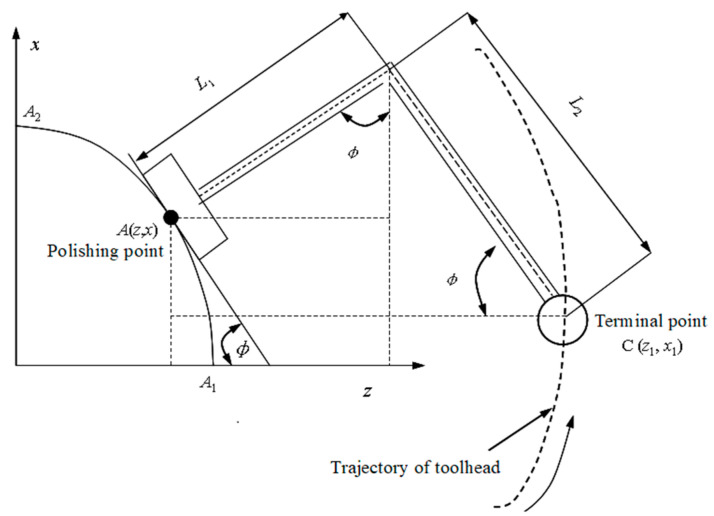
Trajectory of polishing tool.

Based on the polishing system developed by the author, during the polishing process, it is required that the tool axis vector direction of the toolhead at the polishing point is consistent with the normal direction of the aspheric surface, that is, perpendicular to the tangent direction of the aspheric surface, so as to ensure that the polishing force can be applied vertically to the aspheric surface without changing the posture of toolhead. At this time, the posture angle of the toolhead is *θ* = 0. The trajectory of the polishing tool system is shown in Figure 9.

A coordinate system of part is established with the center *O* of the aspheric end face as the origin, and the generatrix equation of part in the *xoz* plane can be expressed as *x* = *f* (*z*) (A_1_A_2_, as shown in Figure 9). The following supposition can be made: *A* (z, x) is the coordinate of the polishing point, and *φ* (acute angle) is the angle between the tangent plane of the part at the polishing point and the z-axis, then tan *φ* = *dx*/*dz*. The terminal point *C* (*z*_1_, *x*_1_) of the tool holder can be seen as the cutter location point of the polishing tool. Its trajectory, which is controlled by the programming of the CNC lathe, can be described as follows:

(26) $$\left\{\begin{array}{l} z_{1}=z+(L_{1})\sin\varphi +L_{2}\cos\varphi \\ x_{1}=x+(L_{1})\cos\varphi -L_{2}\sin\varphi \\ x=f(z)\\ \tan\varphi =f’(z) \end{array}\right.$$

where *L*_1_ is the length from the tool holder to the polishing point.

As analyzed in Section 3.3, during the polishing process, the normal displacement *δ* will be generated among the surface of the polishing head, abrasive particles, and aspheric part. This will cause the actual position of the toolhead on the aspheric surface to change. The position changes in the *z* and *x* directions are Δz = *δ*sin*φ*, Δx = *δ*cos*φ*. In order to ensure that the actual position of the toolhead does not change, it is necessary to compensate for the normal displacement *δ* in the trajectory equation. Then, the trajectory of terminal point C can be written as follows:

(27) $$\left\{\begin{array}{l} z_{1}=z+(L_{1}+\delta )\sin\varphi +L_{2}\cos\varphi \\ x_{1}=x+(L_{1}+\delta )\cos\varphi -L_{2}\sin\varphi \\ x=f(z)\\ \tan\varphi =f’(z) \end{array}\right.$$

Equation (27) is the normal displacement compensation model of the toolhead in the motion space. When the tool holder of the NC lathe is fed according to this trajectory, it can not only ensure that the toolhead feeds perpendicularly to the surface of the aspheric part but also compensate for the position change caused by the normal displacement.

## 5. Experimental Study

### 5.1. Experimental Setup

**Figure 10:**
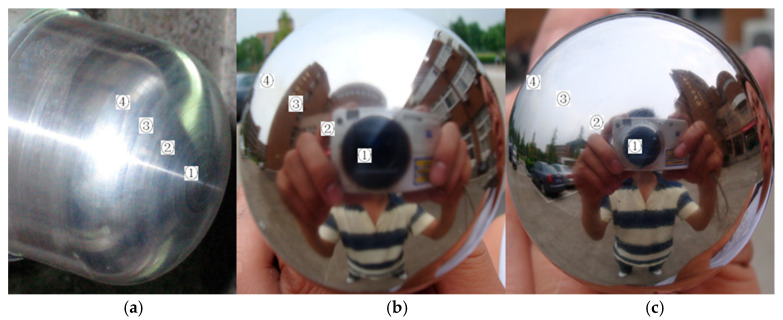
Surface condition of part before and after polished. (**a**) Before polishing; (**b**) polishing with no compensation; (**c**) polishing with compensation.

To verify the validity of the model and method proposed in this paper, polishing experiments were carried out based on the polishing system developed. In order to ensure the initial conditions such as geometry, accuracy, and surface roughness are consistent, two aluminum ellipsoid parts with the same material and the same finish turning were prepared for comparison, which is shown in Figure 10a. The long semi-axis and short semi-axis of the ellipsoid were 23 mm and 15 mm, respectively. Diamond paste with a particle size of 0.25 µm was used. A disc-shaped wool felt was used in the polishing tool with a diameter of 25 mm and a thickness of 8 mm. The surface roughness is measured using the SRM-1 (D) surface roughness measuring instrument. The specific parameters during the polishing process are shown in Table 1.

Two sets of experiments were conducted to verify the above analysis. In the first set, during the polishing process, one part was polished without displacement compensation; that is, the position trajectory of the polishing toolhead adopts Equation (27). In the second experiment, another part was polished using the displacement compensation method. That is, the position trajectory of the polishing toolhead adopts Equation (28). During the two polishing processes, the other polishing parameters, such as the polishing force controlled by MRT, the rotation speed, and the feed rate of the ellipsoid part controlled by the NC lathe, are all the same. The actual polishing force is obtained indirectly by measuring the actual torque output by MRT in the polishing process in real time.

### 5.2. Experimental Results

The experimental results are shown in Figure 10, where Figure 10a shows the aluminum ellipsoid part surface before polishing, Figure 10b shows the part surface after polishing without the normal displacement compensation method, and Figure 10c shows the part surface after polishing with normal displacement compensation method. The surface roughness was measured at four different areas on each part surface. Each different area was measured five times, and the average values were obtained as the roughness value of the measured areas. The measurement results are listed in Table 2.

## 6. Discussion

### 6.1. Surface Roughness

As can be seen from Figure 10b,c. that the mirror-like surfaces are obtained using the polishing experimental system developed in Section 4.1. In the ordinary workshop environment, within 45min, the polishing quality of the aspheric surface can be greatly improved, whether or not the displacement compensation model and method are used. It can be calculated from Table 2 that the average value of surface roughness of parts decreases from 1.645 µm before being polished to 0.04 µm and 0.021 µm after being polished without and with displacement compensation, respectively. The above experimental results provide strong proof of the effectiveness of the developed polishing system. It also can be seen that the value of surface roughness of areas ③ and ④ with displacement compensation is significantly lower than that of no displacement compensation.

At the same time, it should be noted from Table 2 that the roughness values in the area ① of the two parts are quite close for both treatment modes. This is mainly because in area ①, the gyratory radius on the aspheric surface approaches zero, and the rotational speed approaches zero. Although the normal displacement compensation method was used, the material removal rate in this area was also small, resulting in a close roughness value obtained by the two methods.

### 6.2. Unevenness Coefficients

It can be calculated from Table 2 that when the part was polished with the normal displacement compensation method, the unevenness coefficient of surface roughness was 19%, and the other part was 112.5%, which was polished without normal displacement compensation. The unevenness coefficient $\nabla R_{a}$, is expressed as follows:

(28) $${\nabla R}_{a}=\frac{R_{a,max}-R_{a,min}}{{\bar{R}}_{a}}$$

where *R_a,max_*, *R_a,min_*, and ${\bar{R}}_{a}$ are the maximum, minimum, and average values of surface roughness in the four detection areas, respectively.

It is noted that the values of the unevenness coefficient obtained with the normal displacement compensation method are much smaller than those obtained without normal displacement compensation. It is easy to find the reason for using the model and control method proposed in this article; the normal displacement change of each polishing point can be compensated by the motion trajectory of the toolhead. Furthermore, the position error of the polishing point is reduced, and the consistency of surface roughness is improved. Therefore, it can be concluded that more uniform roughness and better surface quality can be obtained using the normal displacement compensation method.

### 6.3. Effect of Particle Shape

In the paper, the normal displacement model considers ideal spherical particles, and a higher surface quality is obtained using a polishing system developed. However, it is well known that the shape and size distribution of particles affect the quality and efficiency of the polishing. Therefore, irregular particles should be discussed.

(1) Material area removal rate. Spherical particles tend to roll more on the part surface, resulting in less material removal. In contrast, irregularly shaped particles, due to their sharp edges or protrusions, are more likely to cut or plow the surface, thus removing material more effectively. It is easier to achieve material removal by using irregular particles than regular abrasives.
(2) Deformation of the part surface. According to Section 3, whether it is spherical particles or irregular particles, the contact area is very small, and the local deformation is minimal. The difference is that spherical particles produce a more uniform stress distribution on the part surface, while irregular particles can generate higher stress concentrations in the contact area, which can easily cause scratches or cracks on the part surface, thus affecting surface quality.

In this paper, the same type of particles produced by the same company were used in the experiment. When the size of the particles is much smaller than the curvature radius of the aspherical surface, it can be considered that the motion behavior of irregular abrasive particles is consistent with that of spherical particles. Of course, the impact of particle shape on the experimental system developed in this paper still needs further experimental verification, which is also one of our future research directions.

## 7. Conclusions

This study has established a model of normal displacement and proposed the compensation method based on a decoupling polishing system. The deformation among the polishing toolhead, abrasive particle, and part has been studied using the Hertz contact theory. The normal displacement model is established. Then, the trajectory of the polishing tool is pre-planned, and the compensation method of normal displacement is proposed based on the polishing system. Experiments were carried out to examine our model and method. The major results of this study are summarized as follows:

(1) When the part was polished without the displacement compensation method, the values of roughness varied greatly with the variation in the curvature radius. The reason is that the normal displacement is mainly affected by the curvature radius of the aspheric surface when the plane toolhead is used. The normal displacement decreases sharply with the increasing curvature radius, and the values of roughness decrease accordingly. This illustrates that displacement compensation is necessary.
(2) The polishing quality can be improved significantly, and the average value of surface roughness of parts is 0.021 µm after being polished with the displacement compensation model and method.
(3) The values of the unevenness coefficient obtained with the normal displacement compensation method are much smaller than those obtained without normal displacement compensation. The results show that the displacement compensation method proposed and the experimental system developed in this paper can achieve a more uniform surface quality and reduce the position error of the toolhead. This further proves that the method can effectively solve the problem of position error control in polishing, which has certain novelty and effectiveness.
(4) The model and method proposed in this paper can effectively achieve the normal displacement compensation, the quality of polishing can be improved greatly, and a more uniform surface quality can be obtained.

Our experimental study confirms that the normal displacement between the toolhead and the part should be compensated during the polishing process when the radius of curvature of the part surface varies. The model and method proposed in this paper can compensate for the normal displacement, and it is a new method for position error control.

## Figures and Tables

**Table 1:** Polishing parameter.

Classification	Diamond Grits	Part	Wool Felt
Elastic modulus *E* (GPa)	1050	70	0.015
Poisson’s ratio *ν*	0.2	0.34	0.079
Brinell hardness *H_B_* (GPa)	102	0.06	-

**Table 2:** Measurement results of surface roughness.

Areas	Roughness Before Polishing *Ra* [µm]	Roughness After Polishing *Ra* [µm]		Polishing Time *t* [min]
		Without Displacement Compensation	With Displacement Compensation	
④	1.82	0.066	0.023	45
③	1.62	0.051	0.021	
②	1.58	0.021	0.019	
①	1.56	0.022	0.020	

## Data Availability

The original contributions presented in the study are included in the article, further inquiries can be directed to the corresponding author.
